# Autocrine effects of PCSK9 on cardiomyocytes

**DOI:** 10.1007/s00395-020-00824-w

**Published:** 2020-11-10

**Authors:** Annemarie Wolf, Hanna Sarah Kutsche, Rolf Schreckenberg, Martin Weber, Ling Li, Susanne Rohrbach, Rainer Schulz, Klaus-Dieter Schlüter

**Affiliations:** Institute of Physiology, Justus-Liebig-University Giessen, Aulweg 129, 35392 Gießen, Germany

**Keywords:** PCSK9, Cardiomyocytes, Cardiac function, oxLDL, Cardiovascular disease

## Abstract

Proprotein convertase subtilisin kexin type 9 (PCSK9) is in the focus of cardiovascular research due to its role in hepatic low density lipoprotein (LDL) clearance. However, extrahepatic expression of PCSK9 such as in cardiomyocytes and its regulation by oxidized LDL (oxLDL) put notion on extrahepatic effects of PCSK9 as well. This study was aimed to reveal the role of PCSK9 in oxLDL-dependent regulation of cardiomyocyte function. Adult rat and mouse ventricular cardiomyocytes and isolated perfused hearts were used. OxLDL was applied to increase PCSK9 expression in cardiomyocytes. Cell function was analyzed by load-free cell shortening as well as left ventricular developed pressure of isolated hearts. OxLDL decreased shortening in wild-type-derived mouse cardiomyocytes but not in those isolated from PCSK9 knockout mice. Overexpression of human PCSK9 in rat cardiomyocytes reduced shortening in the absence of oxLDL. Addition of recombinant PCSK9 mimicked these effects. In cardiomyocytes, oxLDL induced PCSK9 release into the supernatant. Inhibition of PCSK9 by Pep 2–8 or alirocumab attenuated the oxLDL-induced loss of cardiomyocyte shortening. Cardiomyocytes express surfeit locus protein 4 (SURF-4), a protein required for PCSK9 secretion in human embryonic kidney cells (HEK 293 T), and silencing of SURF-4 reduced the oxLDL effects on cardiomyocytes. In isolated perfused rat hearts PCSK9 inhibition by alirocumab improved the function. In addition, left ventricular function of isolated hearts from PCSK9 knockout mice was increased under basal conditions as well as at 10 min and 120 min of reperfusion following 45 min of ischemia. Collectively, the data show that cardiomyocytes express and release PCSK9 that acts in an autocrine way on cardiomyocytes and impairs their function.

**Electronic supplementary material:**

The online version of this article (10.1007/s00395-020-00824-w) contains supplementary material, which is available to authorized users.

## Introduction

Proprotein convertase subtilisin kexin type 9 (PCSK9) acts as a negative regulator of the low-density lipoprotein (LDL) receptor in the liver and therefore increases serum LDL cholesterol (LDL-C) [13]. Since hypercholesterolemia is still one of the main risk factors for cardiovascular disease (CVD) [22] and CVD mortality [1], PCSK9 evolved as a new target in the treatment of hypercholesterolemia [6].

Given that subjects with a loss of function mutation of PCSK9 gene show an additional cardiovascular protection independent of LDL-C lowering [4, 7, 19], a different role of PCSK9 in the cardiovascular system has been suggested. Whereas distinct extrahepatic functions of PCSK9 have already been described [31], a direct effect of PCSK9 on cardiac function is still a matter of debate [3].

Previously, we could demonstrate that terminally differentiated ventricular rat cardiomyocytes constitutively express PCSK9 [28]. Furthermore we found that oxLDL, which also plays a pivotal role in the pathophysiology of cardiovascular disease [11, 35], impaired cardiomyocyte function in a PCSK9-dependent way. However, the mechanism by which PCSK9 is involved in this process remained elusive. Nevertheless, oxLDL increased the expression of PCSK9 in cardiomyocytes and silencing of PCSK9 in cardiomyocytes attenuated oxLDL-dependent effects on cell shortening [28]. This might indicate either, that intracellular PCSK9 affects cell function or that cardiomyocytes release PCSK9 which then affects the function of cardiomyocytes.

Therefore, we used isolated cardiomyocytes from rats and mice to clarify whether modification of intracellular or extracellular PCSK9 is involved in oxLDL effects on cardiomyocyte and/or cardiac function.

## Material and methods

### Materials

OxLDL (human high oxidized low density lipoprotein), Low-oxidized LDL and LDL was produced by KB Kalen Biomedical, Montgomery Village, USA and purchased from Biotrend Chemikalien, Cologne, Germany. OxLDL stock solution was stored at 4 °C and used within six weeks. Human recombinant PCSK9 (hPCSK9) was purchased from Cayman Chemical, Michigan, USA. Malondialdehyde-modified human serum albumin (MDA-HSA) was produced by Cell Biolabs, Inc., California, USA and purchased from Biotrend Chemikalien.

### Animals

Adult Wistar rats (175–225 g) and C57BL6/JR (25–35 g) were ordered from Janvier Labs (Le Genest Saint Isles, France). PCSK9 knockout mice (B6;129S6-Pcsk9^tm1Jdh^(PCSK9^−/−^), 25–30 g) and appropriate controls (B6129SF2/J (PCSK9^+/+^), 8–9 weeks old) were supplied from The Jackson Laboratory (Maine, USA) with consent of the University of Texas Southwestern Medical Center. Mice and rats were housed according to the Guide for the Care and Use of Laboratory Animals (NIH Publication No. 85–23, revised 1996). All protocols were approved by the Justus-Liebig-University Giessen (permission number: 666_M and 561_M). In this study only male animals were used, to avoid the effect of sex on experiments.

### Isolation and cultivation of cardiomyocytes

Ventricular heart muscle cells were isolated from rats and mice as described previously [5, 25]. In brief, rats or mice have been sacrificed under deep anesthesia (isoflurane 5%) before the heart was excised and transferred to ice-cold saline. Thereafter the aorta was cannulated and the heart was connected to a Langendorff perfusion system where it was perfused with Powell medium (NaCl 110 mM, KCl 2.5 mM, KH_2_PO_4_ 1.2 mM, MgSO_4_ × 7H_2_O 1.2 mM, Hepes 25 mM, D( +)-glucose-monohydrate 10 mM, pH adjusted to 7.4), that contained collagenase (25 mg/ml, 265 u/mg, Worthington Biochemicals, Lakewook, USA) and CaCl_2_ (25 µM), for 25 min at 37° C. Thereafter, ventricular tissue was minced and incubated in the abovementioned solution for another 5 min at 37° C. The remaining cell solution was filtered through a 200-µm nylon mesh. The filtered material was resuspended in Powell medium, centrifuged and after a stepwise increase in calcium concentration finally transferred to culture medium (Medium 199, supplemented with creatine, carnitine, taurine, and 2% penicillin–streptomycin, Biochrom). Cells were plated onto culture dishes (35 mm) which were precoated with 4% (*v*/*v*) fetal calf serum (FCS) (PAA, BioPharm). After one hour cell culture medium was refreshed and PCSK9 (200 ng/ml), oxLDL (5–20 µM), Pep 2–8 (10 µM), alirocumab (1.5 mg/ml) or combinations thereof were added (as indicated) to the culture medium. Cells were then cultured under serum-free conditions for 24 h at 37° C in the above mentioned medium. Where indicated, small interfering RNA (siRNA) directed against SURF-4 (final concentration 0.05 µM) was added 6 h before administration of oxLDL (5–20 µM) to the culture medium. SiRNA was purchased from Qiagen, Venlo, Netherland.

### Adenoviral infection of cardiomyocytes and HepG2 cells

Isolated cardiomyocytes were infected with hPCSK9. Therefore an adenoviral plasmid with the insert of hPCSK9 gain of function mutation D374Y (pAd/CMV/V5 hPCSK9, Addgene, Massachusettes, USA) was generated by gateway cloning [15, 17] (pENTR™ Directional TOPO®Cloning Kit,Thermo Fisher Scientific, Massachusettes, USA). An adenoviral plasmid with the insert of LacZ was generated for control (pAd/CMV/V5 LacZ, Addgene, Massachusettes, USA). HEK 293 A cells have then been transfected with these adenoviral plasmids (hPCSK9, LacZ). The adenovirus produced by HEK 293 A cells was collected and used to infect adult ventricular cardiomyocytes as well as HepG2 cells with hPCSK9 and LacZ, respectively. The supernatants of HepG2 cells that contained hPCSK9 or LacZ were collected 72 h after infection, centrifuged (6000×*g*) and then stored at − 80 °C. Cardiomyocytes have been infected with hPCSK9/LacZ or incubated with supernatants of HepG2 cells over 24 h before load-free cell shortening was determined.

### Cell shortening

Cells were stimulated via two AgCl electrodes with biphasic electrical stimuli composed of two equal but opposite rectangular 50 V stimuli of 5 ms duration as described before [18]. Cells were stimulated at 2 Hz frequency. Four signals were registered from each cell. The mean of these four measurements was used to define the contractile responsiveness of a given cell. Cell lengths were measured at a rate of 500 Hz via a line camera. Cells were used in M199 with an extracellular calcium concentration of 1.25 mM. Data are expressed as ΔL/L (%) in which the shortening amplitude (ΔL) is expressed as percent of the diastolic cell length (L). Furthermore, maximal contraction and relaxation velocity (µm/s) were analyzed.

### ELISA

Release of PCSK9 into the supernatant from cardiomyocytes as well as from hPCSK9 overexpressing HepG2 cells was quantified by the use of a commercial available kit purchased by Cusabio Biotech Co. China (Rat PCSK9 ELISA kit) and Cloud-Clone Corp. Texas, USA (Human PCSK9 ELISA).

### Langendorff perfusion of hearts isolated from rat our mouse

The left ventricular function of isolated rat hearts was evaluated as described before [30]. Therefore hearts were excised as mentioned above and attached to a 16-gauge needle. The cannulated heart was then connected to a Langendorff perfusion system where it was perfused retrograde with an oxygenated saline buffer (NaCl 140 mM, NaHCO_3_ 24 mM, KCl 2.7 mM, NaH_2_PO_4_ 0.4 mM, MgCl_2_ 1 mM, CaCl_2_ 1.8 mM and glucose 5.0 mM, pH 7.4). A polyvinylchloride balloon, which was connected to a pressure transducer, was carefully inserted into the left ventricle to assess left ventricular pressure (LVDP), dp/dt max as well as dp/dt min.

To validate a possible function of PCSK9 on left ventricular function isolated hearts were perfused with a monoclonal antibody against PCSK9 (alirocumab used in a concentration of 750 µg).

In addition hearts from PCSK9 knockout and control mice were prepared and attached to an Aortic Cannula (Ø 1 mm, Hugo Sachs Elektronik-Harvard Apparatus, March, Germany). The cannulated heart was then connected to a Langendorff perfusion system (Hugo Sachs Elektronik-Harvard Apparatus, March, Germany) where it was perfused retrograde with a 37 °C warm modified Krebs Henseleit buffer (NaCl 118 mM, KCl 4.7 mM, MgSO_4_ 0.8 mM, KH_2_PO_4_ 1.2 mM, G/glucose 5 mM, CaCl_2_ 2.5 mM, NaHCO_3_ 25 mM, pyruvate 1.9 mM; pH 7.4; sterile-filtered with an 0.2 µm filter; saturated with 95% oxygen and 5% carbogen). The perfusion pressure was adjusted to 70 mmHg (transduced by a Replacement Transducer Head for APT300 Pressure Transducer, Hugo Sachs Elektronik-Harvard Apparatus) and kept constant during the whole experiment. A small ballon (built up from a cling-film), which was connected to a pressure transducer (Combitrans 1-fach Set Mod.II University Giessen, B. Braun, Melsungen, Germany), was carefully inserted into the left ventricle and inflated up to 12–14 mmHg (end diastolic pressure) to assess left ventricular function. Additionally the hearts were paced at 600 bpm to ensure a stable heart rate. Left Ventricular Pressure (LVPD = systolic pressure-diastolic pressure) was determined as well as dLVP/dt_max_ and dLVP/dt_min_. For the ischemia/reperfusion protocol: After stabilization phase (5 min) the perfusion and pacing was switched off for 45 min to generate a no flow ischemia. Thereafter the heart was reperfused for another 120 min.

### RNA Isolation and qRT-PCR

RNA was isolated from liver samples and isolated cardiomyocytes using peqGold Trifast (peqlab, Biotechnologie GmbH, Erlangen, Germany) according to manufacturer’s protocol. To avoid genomic DNA contamination, samples were treated with DNase (1U/ µg RNA; Invitrogen, Karslruhe, Germany) for 15 min at 37 °C. The Reverse Transcription of RNA (1 µg/10 µl) into cDNA was performed with Superscript RNase reverse transcriptase (200 U/µg RNA; Invitrogen) and oligo dTs at 37 °C for 60 min. Quantitative Real-Time PCR was performed using the Icycler MyiQ® detection systems (Bio-Rad, Munich, Germany) in combination with IQ SYBR green real-time supermix (Bio-Rad, Munich, Germany). Primer for surfeit locus protein-4 (SURF-4) and beta-2-microglobulin (B2M) (Table 1) were used and quantification was performed applying the ΔΔ Ct method [20].

**Table 1:** List of primer used in this study

Gene	Forward	Reverse
B2M	GCCGTCGTGCTTGCCATTC	CTGAGGTGGGTGGAACTGAGAC
SURF-4	ATTTCGCCGACCAGTTCCTT	CAGGTAACCACAGCTCCAGG
PCSK9 (human)	CACCATGGGCACCGTCAGCTCCAG	AAACTGGAGCTCCTGGGAGGCC
PCSK9 (mouse)	TTGAACAAACTGCCCATCGC	CCCAACAGGTCACTGCTCAT

### Western Blot

Isolated cardiomyocytes were incubated with cell lysis buffer (Cell Signaling, Technology, Frankfurt, Germany) as described before [29]. Samples were treated 1:1 with Leammli buffer (Sigma-Aldrich, Taufkirchen, Germany) and loaded (~ 100 µg protein) on a 10% SDS-PAGE (Life Technology, Darmstadt, Germany). Following electrophoresis proteins were subsequently transferred to a nitrocellulose membrane. Anti-PCSK9 polyclonal antibody produced in goat (#AF3985, R&D Systems, Minnesota, USA) was used to detect the expression of PCSK9 protein. Glyceraldehyde 3-phosphate dehydrogenase (GAPDH) served as loading control. Detection of GAPDH was performed using anti-GAPDH monoclonal antibody produced in mouse (#CB1001, Calbiochem®, Schwalbach, Germany). Secondary antibodies were purchased from Dako (now Agilent Technologies, Santa Clara, USA).

### Statistics

Data are expressed as means ± SD, indicated in the legend of the figures. The statistical comparison of two groups was performed by two-sided-*t*-test, if normal distribution of samples could be verified, otherwise Mann–Whitney-*U* test was applied. Comparison of more than two groups was performed with one-way following a post hoc analysis with the Student–Newman–Keuls test. *p* levels ≤ 0.05 were regarded as significant and indicated as an asterisk or as indicated in the figure’s legend. Figures were generated with Graph Pad Prism 8.

## Results

### OxLDL-induced reduction of cardiomyocyte cell-shortening requires PCSK9

**Fig. 1:**
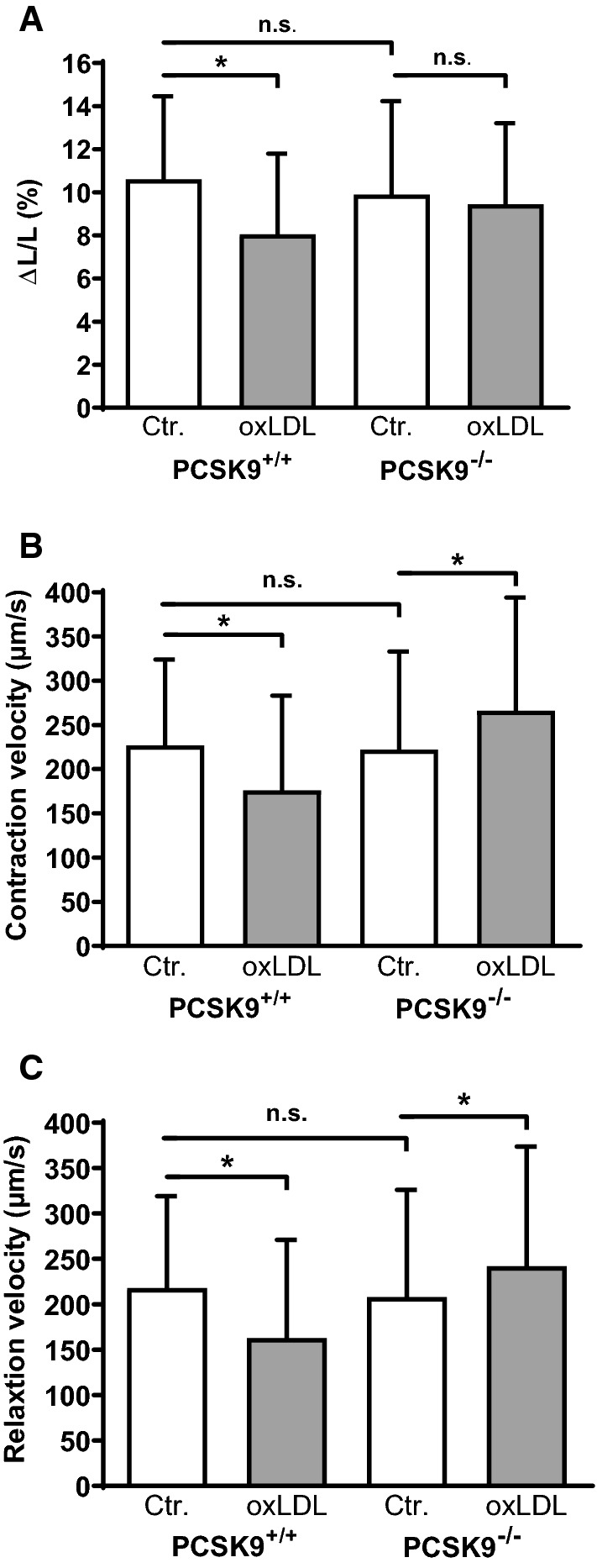
Effect of oxLDL on load-free cell shortening of isolated cardiomyocytes derived from PCSK9^−/−^- and PCSK9^+/+^-mice. Serum-free cultured adult ventricular cardiomyocytes were exposed to 5 µM oxLDL for 24 h. Load free cell shortening (cells were paced at 2 Hz) is expressed (**a**) as ΔL/L (%), (**b**) contraction velocity (µm/s) (**c**) and relaxation velocity (µm/s) of 222 (PCSK9^+/+^, Control), 126 (PCSK9^+/+^, oxLDL), 162 (PCSK9^−/−^, Control) and 134 (PCSK9^−/−^, oxLDL) cells (14–25 independent experiments with an intraassay variability of *p* > 0.05). Statistical analysis was performed by Mann–Whitney test. *p ≤ 0.05, *n.s.* not significant. Data are mean ± SD

To investigate whether endogenously expressed PCSK9 alters cell shortening of adult ventricular cardiomyocytes, we isolated adult ventricular cardiomyocytes from PCSK9^−/−^- as well as PCSK9^+/+^-mice. Comparison between both strains revealed no obvious difference (Fig. 1). Cardiomyocytes from both strains were cultivated under serum-free conditions and also incubated for 24 h with oxLDL (5 µM). Thereafter function was measured by load-free cell shortening. While the incubation with oxLDL (5 µM) led to a significant decrease of relative cell shortening (Fig. 1a) as well as contraction (Fig. 1b) and relaxation velocities (Fig. 1c) in PCSK9^+/+^-cardiomyocytes, this effect was absent in cardiomyocytes isolated from PCSK9^−/−^-mice (Fig. 1a–c). Instead, there was an increase in contraction and relaxation velocity in cardiomyocytes that lacked PCSK9 in the presence of oxLDL (Fig. 1b + c).

### Effect of PCSK9 overexpression on load free cell shortening of cardiomyocytes

**Fig. 2:**
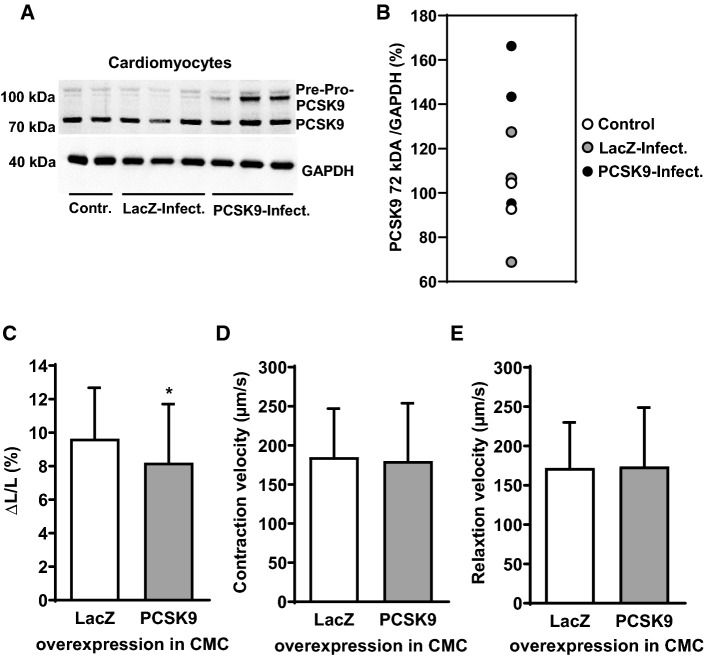
Infection of cardiomyocytes with human PCSK9. Serum-free cultured adult rat ventricular cardiomyocytes were infected with hPCSK9. Infection of cardiomyocytes with LacZ served as control. After 24 h, load free cell shortening was measured (cells paced at 2 Hz). **a** Representative immunoblot indicating the successful transfection of cardiomyocytes with hPCSK9. **b** Quantification of Western Blot shown in (**a**). **c** Load free cell-shortening expressed as ΔL/L (%), (**d**) contraction velocity (µm/s) (**e**) and relaxation velocity (µm/s) of 89 (LacZ) and 90 (PCSK9) cells (10 independent experiments with intraassay variability *p* > 0.05). Statistical analysis was performed by Mann–Whitney test. *p ≤ 0.05. Data are mean ± SD

Given the above findings we hypothesized that PCSK9 affects cardiomyocyte function independent from oxLDL-stimulation. Adult ventricular cardiomyocytes (from rats) were infected with an adenovirus bearing hPCSK9 for 24 h to induce PCSK9 overexpression in cardiomyocytes. The control group was infected with an adenovirus bearing LacZ. To proof the successful transfection of cardiomyocytes, the protein expression of PCSK9 was analyzed by Western Blot (Fig. 2a). In the lysates of cardiomyocytes that had been infected with hPCSK9 a more prominent band of pre-pro-PCSK9 (~ 100 kDa) as well as of the ~ 72 kDa form of PCSK9 appeared, that were less intense in controls as well as in cardiomyocytes infected with LacZ (Fig. 2a + b). After 24 h of adenoviral infection cardiomyocyte function was measured by load-free cell shortening (Fig. 2c, d). While relative cell shortening (Fig. 2c) was significantly decreased in cardiomyocytes that had been infected with hPCSK9, neither relaxation velocity (Fig. 2e) nor contraction velocity (Fig. 2d) were impaired due to the infection with hPCSK9 (*p ≤ 0.05).

### Effect of increased extracellular PCSK9-levels on cardiomyocyte function

**Fig. 3:**
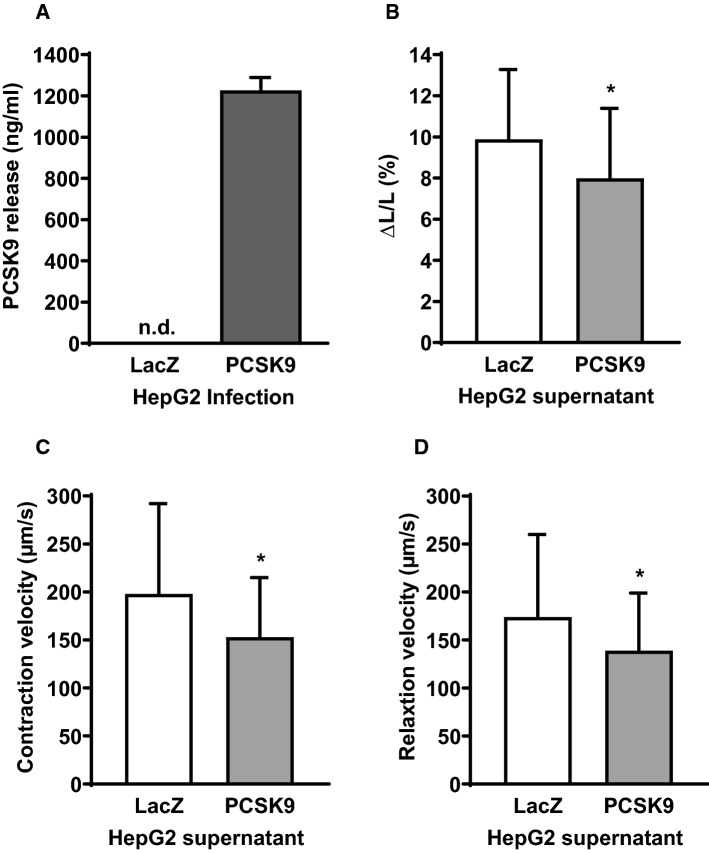
Effect of hPCSK9 incubation on cell shortening of cardiomyocytes. Serum-free cultured adult rat ventricular cardiomyocytes were incubated for 24 h with supernatants derived from HepG2 cells that have been infected with hPCSK9 or LacZ for 72 h. **a** PCSK9 concentration in supernatant of HepG2 cells infected with LacZ or PCSK9 was determined by ELISA (*n* = 4). **b** Load free cell shortening is expressed as ΔL/L (%), (**c**) contraction velocity (µm/s) (**d**) and relaxation velocity (µm/s) of 160 (LacZ) and 180 (PCSK9) cells (14 independent experiments with intraassay variability of *p* > 0.05). Cells were paced at 2 Hz. Statistical analysis was performed by Mann–Whitney test. *p ≤ 0.05. Data are mean ± SD

To distinguish between the effect of PCSK9 overexpression and an increase in extracellular PCSK9 on cardiomyocyte function, we incubated adult rat ventricular cardiomyocytes with supernatants derived from PCSK9 or LacZ overexpressing HepG2 cells. Therefore HepG2 cells were infected with hPCSK9 or LacZ. Cardiomyocytes were then incubated with PCSK9 (122.64 ± 6.23 ng/ml, *n* = 4) or LacZ containing supernatants for 24 h before load-free cell shortening was measured (Fig. 3). The incubation of cardiomyocytes with HepG2 supernatants containing elevated levels of PCSK9 led to a significant decrease of cell shortening compared to the presence of LacZ enriched supernatants (Fig. 3b). Furthermore contraction velocity (Fig. 3c) as well as relaxation velocity (Fig. 3d) were significantly reduced in the presence of HepG2 derived PCSK9-supernatants (*p ≤ 0.05).

### Effects of recombinant PCSK9, Pep 2–8 and alirocumab on cardiomyocyte function

**Fig. 4:**
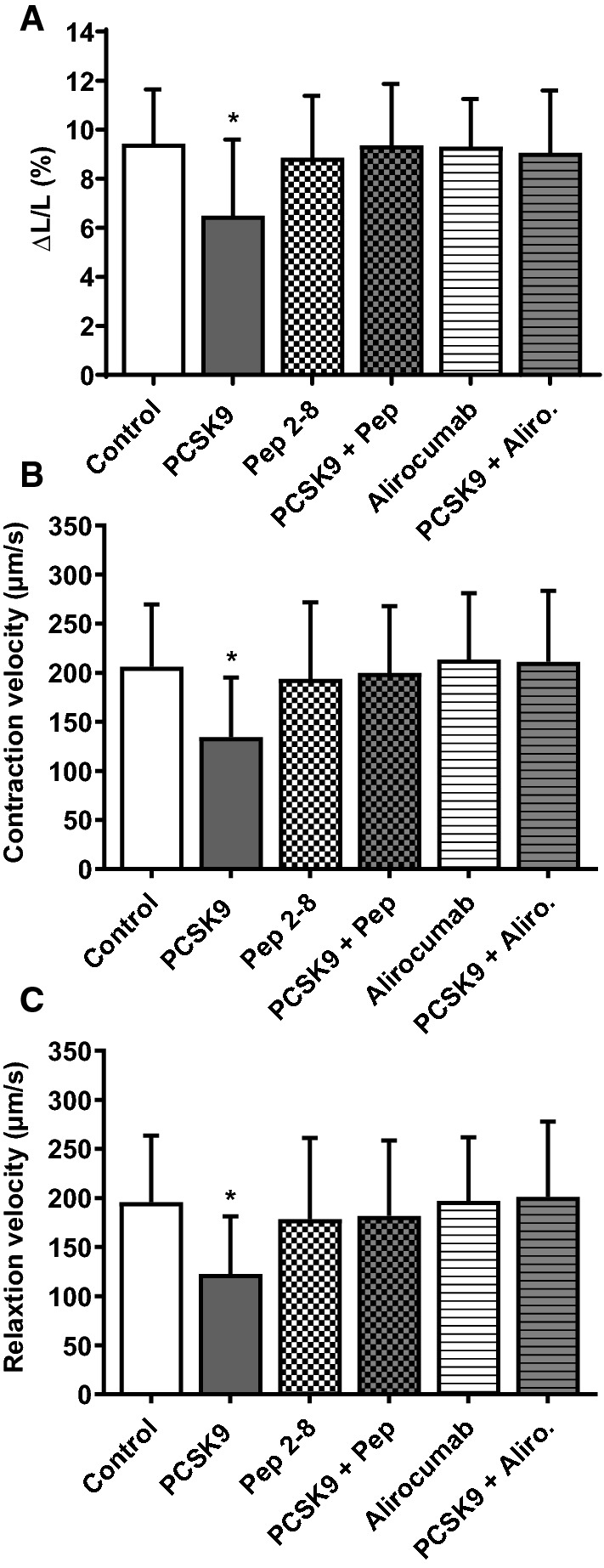
Effect of recombinant PCSK9 on cell shortening in the presence of Pep 2–8 or alirocumab. Adult rat ventricular cardiomyocytes were cultured under serum-free conditions and incubated with recombinant PCSK9 (200 ng/ml), the PCSK9 inhibitor Pep 2–8 (10 µM), the monoclonal PCSK9 antibody alirocumab (1.5 mg/ml) and combinations thereof. After 24 h load free cell shortening was measured (cells were paced at 2 Hz) and expressed as (**a**) ΔL/L (%), (**b**) contraction velocity (µm/s) and **c** relaxation velocity (µm/s) of Control = 278, PCSK9 = 262, Pep 2–8 = 114, PCSK9 + Pep = 111, Alirocumab = 147 and PCSK9 + Aliro. = 48 cells (5–20 independent experiments with intraassay variability of *p* > 0.05). Statistical analysis was performed by one-way ANOVA and Student–Newman–Keuls for post hoc analysis. *p ≤ 0.05 vs. Control, Pep 2–8, PCSK9 + Pep, Alirocumab and PCSK9 + Aliro. Data are mean ± SD

To validate the effects of extracellular PCSK9 on cardiomyocyte function and exclude any unspecific effect of HepG2 supernatants we incubated cells with purified recombinant PCSK9 for 24 h. At first, a concentration-effect curve was performed to identify the most effective concentration (2 µg/ml, 200 ng/ml and 20 ng/ml) of PCSK9. After 24 h function was determined by load-free cell shortening. Relative cell shortening in these cells was reduced from 9.81 ± 3.12% to 6.29 ± 3.43% (2 µg/ml), 7.76 ± 3.99% (200 ng/ml) and 8.78 ± 3.56% (20 ng/ml) respectively (*n *= 54 cells). In all further experiments a PCSK9 concentration of 200 ng/ml was applied. In the presence of recombinant PCSK9 a significant decline in cell shortening (Fig. 4a) as well as contraction (Fig. 4b) and relaxation velocities (Fig. 4c) in adult ventricular cardiomyocytes (*p ≤ 0.05) was observed. In addition, the PCSK9 inhibitor Pep 2–8 (10 µM) and the monoclonal antibody against PCSK9 (alircoumab, Sanofi, 1.5 mg/ml) antagonized the negative effect of PCSK9 on cellular function, while neither Pep 2–8, nor alirocumab had any effect on basal cardiomyocyte function, expressed as relative cell shortening (Fig. 4a), contraction (Fig. 4b) and relaxation velocities (Fig. 4c).

### OxLDL-induced reduction of cell shortening is accombined by the release of PCSK9 from cardiomyocytes

**Fig. 5:**
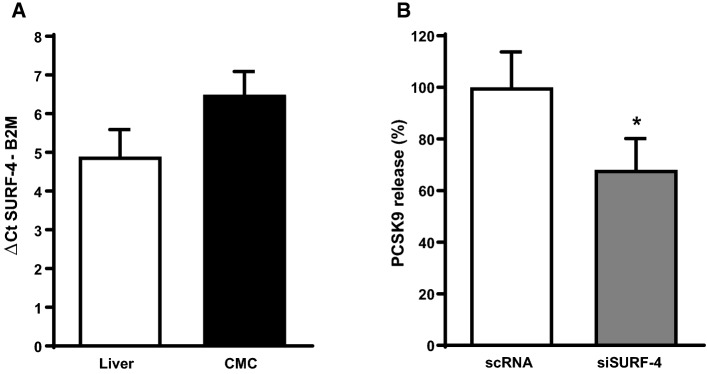
Silencing of SURF-4 in cardiomyocytes affects PCSK9 release. **a** Expression of SURF-4 in liver and cardiomyocytes (CMC) of adult Wistar rats. Threshold cycle (Ct) of SURF-4 amplification, normalized to the mean Ct of B2M-amplification (ΔCt). *n* = 3 (liver) and *n* = 10 (CMC). **b** Adult ventricular cardiomyocytes were cultivated under serum-free conditions for 24 h in the presence of scrambled RNA (*n* = 4) or siRNA targeted against SURF-4 (*n* = 5). PCSK9 release from cardiomyocytes was analyzed by ELISA and normalized to controls (scRNA). Statistical analysis was performed by unpaired *t* test. *p ≤ 0.05. Data are mean ± SD

**Fig. 6:**
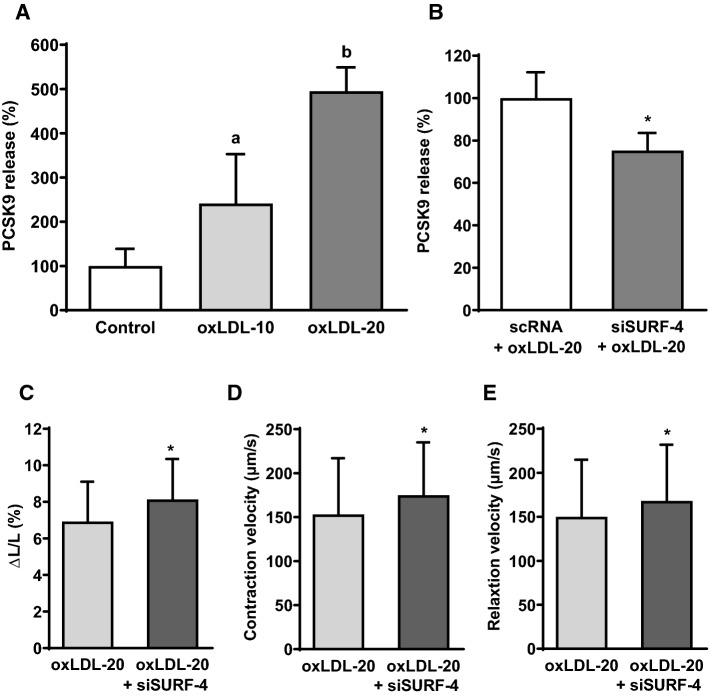
Participation of SURF-4 on the oxLDL-dependent effect on load free cell shortening of cardiomyocytes. **a** Adult rat ventricular cardiomyocytes were cultured under serum-free conditions and exposed to 10 or 20 µM oxLDL. After 24 h PCSK9 concentration (pg/ml) in the supernatant of these cells was analyzed via ELISA and normalized to controls, expressed as PCSK9 release (% of control). Data are mean ± SD from *n* = 10 (control), *n* = 17 (oxLDL-10) and *n* = 4 (oxLDL-20) independent experiments. Statistical analysis was performed by one-way ANOVA and Student–Newman–Keuls for post hoc analysis. *a* < 0.05 vs. control and oxLDL-20, *b* < 0.05 vs. control and oxLDL-10. **b** PCSK9 release from cardiomyocytes (CMC) was quantified by ELISA. Cells were cultivated serum-free and in the presence of scrambled RNA (*n* = 4) or siRNA against SURF-4 (50 nM) (*n* = 5) in combination with oxLDL (20 µM) for 24 h. Statistical analysis was performed by unpaired t-test. *p < 0.05. **c** Load free cell shortening of adult rat cardiomyocytes that were incubated for 24 h with oxLDL (20 µM) and oxLDL (20 µM) + siRNA against SURF-4 (50 nM). Expressed as ΔL/L (%) as well as (**d**) contraction velocity (µm/s) and **e** relaxation velocity (µm/s). *n* = 110 and 101 cells (10–12 independent experiments with an intraassay variability of *p* > 0.05). Statistical analysis was performed by unpaired t-test for (**c**) as well as Mann–Whitney test for (**d**) and (**e**). *p ≤ 0.05. Data are mean ± SD

To evaluate if oxLDL might induce PCSK9 release from cardiomyocytes we measured the PCSK9 concentration in supernatants of cardiomyocytes that had been incubated with oxLDL (10 or 20 µM) for 24 h (Fig. 6a). There was a concentration-dependent increase of PCSK9 in the supernatants of cardiomyocytes. The mean PCSK9 concentration in supernatants of controls was 565.8 ± 219.7 pg/ml. As SURF-4 is known to play a crucial role in secretion of PCSK9 in HEK239 cells [10] we screened adult ventricular cardiomyocytes for SURF-4 expression by qPCR. In cardiomyocytes (CMC), the threshold cycle (Ct) of SURF-4 amplification (normalized to the Ct of B2M amplification) (Fig. 5a) was 6.49 ± 0.6 while ΔCt of SURF-4 amplification in liver samples was 4.89 ± 0.07. To test if the oxLDL-induced increase in PCSK9 release and therefore the impaired cardiomyocyte function might require SURF-4, we silenced SURF-4 by administration of siRNA (SURF-4 mRNA in cardiomyocytes was reduced to 69.75 ± 21.83% of controls by incubation with siRNA) prior to incubation with oxLDL. Silencing of SURF-4 resulted in a decreased release of PCSK9 from cardiomyocytes (Fig. 5b) under basal conditions as well as in the presence of oxLDL (20 µM) (Fig. 6b).

In addition silencing of SURF-4 increased cell shortening (expressed as ΔL/L (%), contraction and relaxation velocity (µm/s)) of cardiomyocytes that showed reduced function due to incubation with oxLDL (Fig. 6c–e).

### Effects of oxLDL on cardiomyocyte cell shortening in the presence of Pep 2–8 or alirocumab

**Fig. 7:**
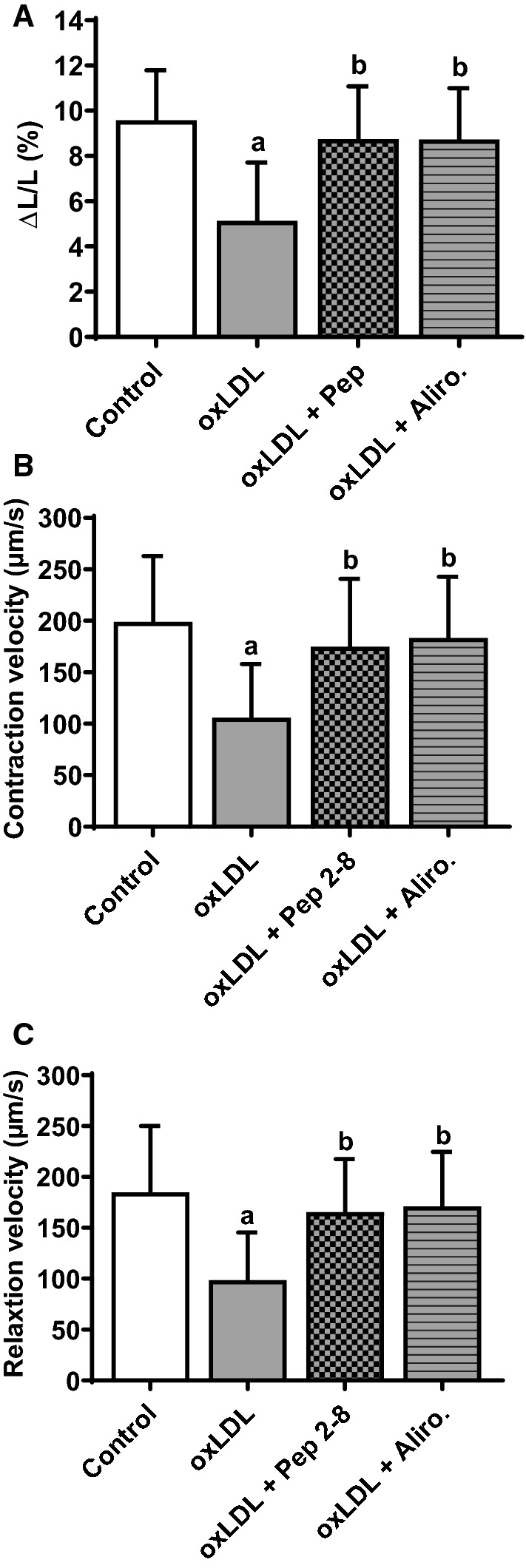
Effect of oxLDL on cell shortening in the presence of Pep 2–8 and alirocumab*.* Adult rat ventricular cardiomyocytes were cultured under serum-free conditions and incubated with oxLDL (20 µM), and combinations of oxLDL with the PCSK9 inhibitor Pep 2–8 (10 µM) or the monoclonal PCSK9 antibody alirocumab (1.5 mg/ml) respectively. After 24 h, load free cell shortening was determined (cells were paced at 2 Hz) and is expressed as (**a**) ΔL/L (%), (**b**) contraction velocity (µm/s) and **c** relaxation velocity (µm/s) of Control = 198, oxLDL = 198, oxLDL + Pep = 90 and oxLDL + Aliro. = 142 cells (16–22 independent experiments with an intraassay variability of *p* > 0.05). Statistical analysis was performed by one-way ANOVA and Student–Newman–Keuls for post hoc analysis. *a* ≤ 0.05 vs. Control, oxLDL + Pep 2–8 and oxLDL + Aliro., *b* ≤ 0.05 vs. oxLDL and Control. Data are mean ± SD

To address the question if oxLDL leads to an increased release of PCSK9 from cardiomyocytes and therefore to a reduction of cardiomyocyte function, we incubated isolated cardiomyocytes with oxLDL (20 µg/ml) and oxLDL in combination with Pep 2–8 (10 µM) or alirocumab (1.5 mg/ml). After 24 h cell function was measured by load-free cell shortening (Fig. 7a–c). While oxLDL led to a significant (*p ≤ 0.05) decrease of relative cell shortening (Fig. 7a), as well as contraction (Fig. 7b) and relaxation velocities (Fig. 7c), the oxLDL effect on cardiomyocyte function was strongly reduced when incubating the cells concurrently with Pep 2–8 or alirocumab. Both PCSK9 antagonists had no effect on cardiomyocyte function per se (Fig. 4a–c). We further evaluated if the effect using oxLDL depends on the level of oxidation. Therefore we measured load-free cell shortening of cardiomyocytes incubated for 24 h with non-oxidized LDL, low oxidized LDL, or high oxidized LDL. Neither LDL nor low oxidized LDL had an effect on load free cell shortening of cardiomyocytes at a concentration at which high oxidized LDL already depressed cell function (Supp. Figure 1B). Also an oxidatively modified non-lipid plasma protein, such as MDA-modified HSA, had no effect on cell-shortening (Supp. Figure 1A).

### Alirocumab affects left ventricular function of isolated perfused hearts

**Fig. 8:**
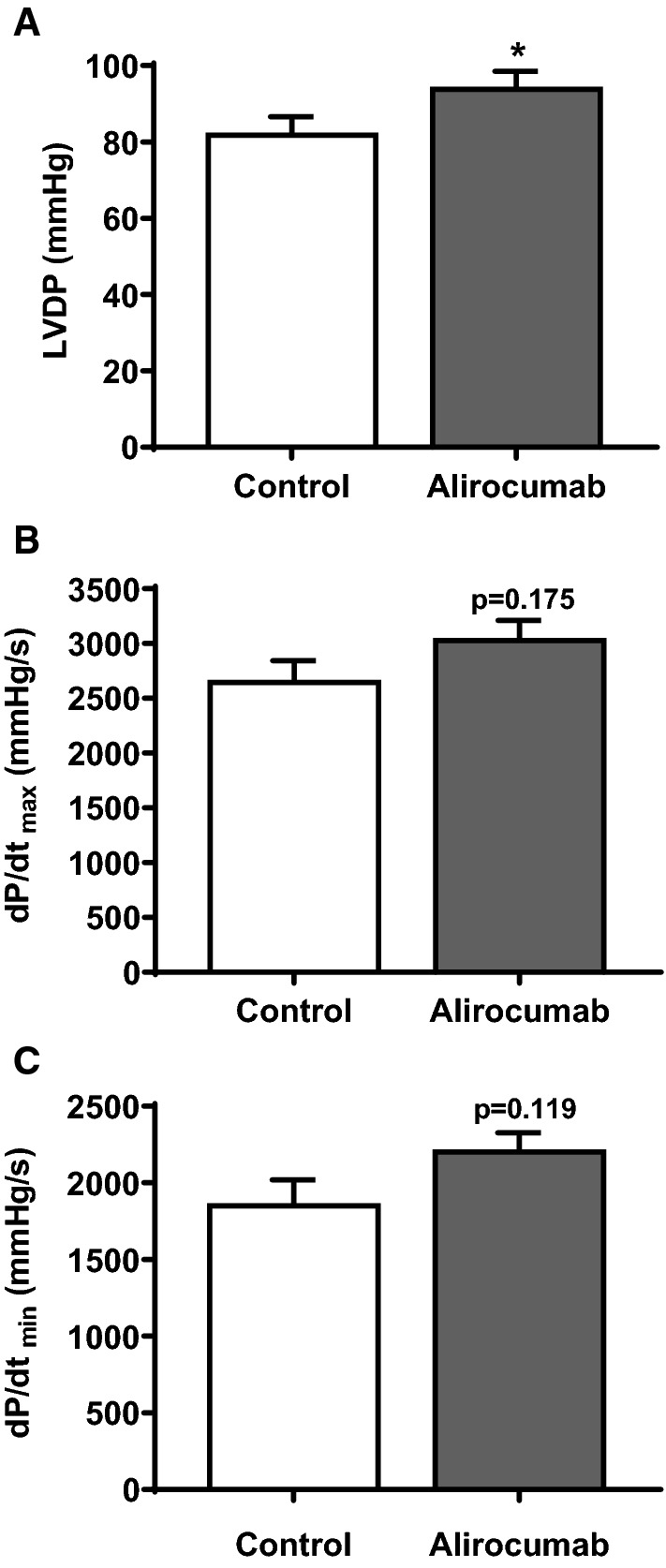
Effect of PCSK9 antibody alirocumab on basal cardiac function of isolated perfused rat hearts*.* Hearts from adult Wistar rats were isolated and transferred to a Langendorff perfusion system. Control hearts were perfused with pure buffer while another group was perfused with buffer containing alirocumab (750 µg/ml). **a** Left ventricular developed pressure (LVDP) in mmHg, **b** dP/dt_max_ (mmHg/s) and **c** dP/dt_min_ (mmHg/s) each out of *n* = 6 are shown as mean ± SD. Statistical analysis was performed by unpaired *t* test. *p ≤ 0.05

Finally, we investigated the effect of alirocumab on basal left ventricular function to exclude that the observed cardiomyocyte effects of PCSK9 can be neglected in vivo. Isolated hearts from adult rats were used and left ventricular function was assessed. Mean PCSK9 concentration in the perfusates collected during the stabilization phase was 1443 ± 885 pg/ml (*n* = 6). Addition of alirocumab (750 µg/ml) to the perfusion buffer (for 10 min) increased left venctricular developed pressure (LVDP, mmHg) (Fig. 8a) as well as dP/dt_max_ (Fig. 8b) (mmHg/s) and dP/dt_min_ (mmHg/s) (Fig. 8c).

### PCSK9 knockout improves left ventricular function of isolated mice hearts basal and during reperfusion

**Fig. 9:**
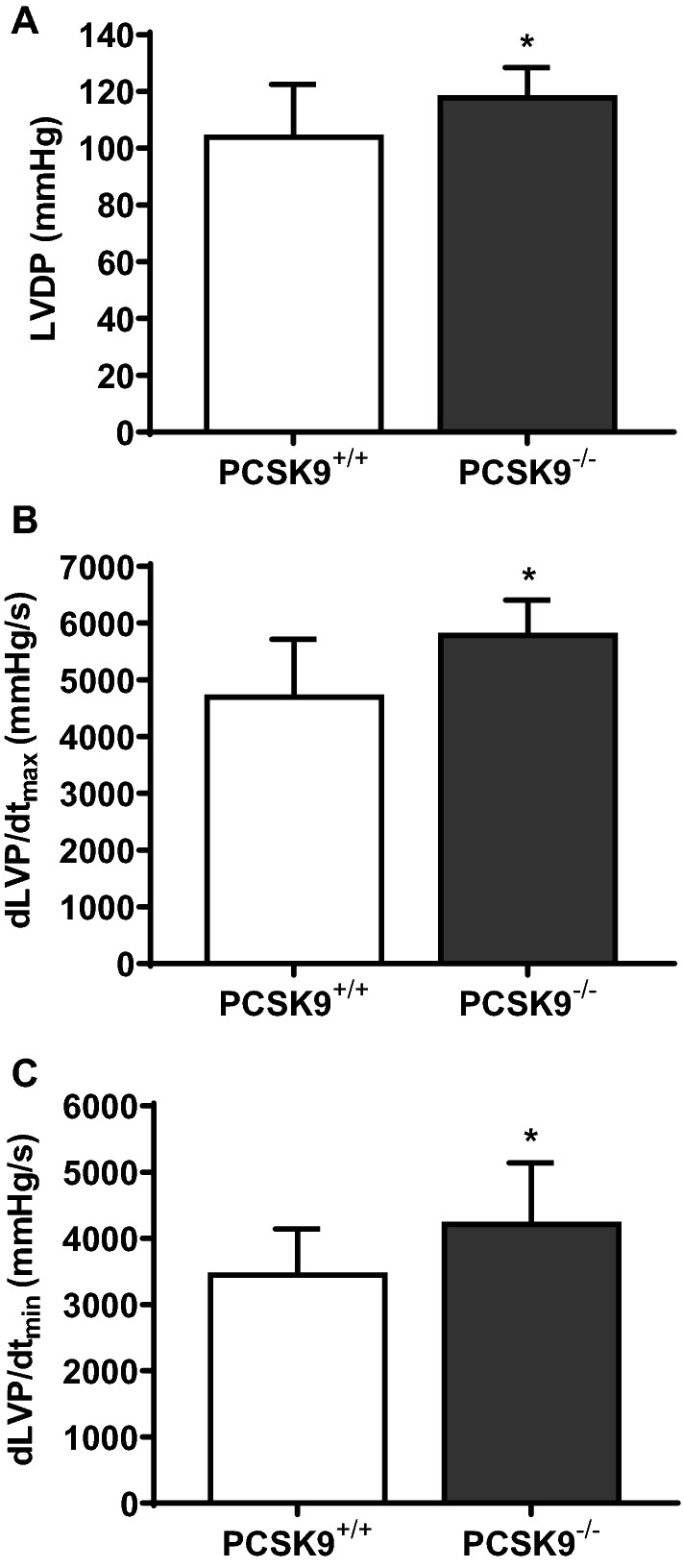
Effect of PCSK9 knockout on basal cardiac function of isolated and perfused mice hearts. Hearts from PCSK9^−/−^-mice were isolated and transferred to a Langendorff perfusion system. **a** Left ventricular developed pressure (LVDP) in mmHg (*n* = 12), **b** dLVP/dt_max_ (mmHg/s) (*n* = 13) and **c** dLVP/dt_min_ (mmHg/s) (*n* = 13) (at the beginning of the stabilization phase) are shown as mean ± SD. Statistical analysis was performed by unpaired *t*-test for (**a**) and (**b**) as well as Mann–Whitney test for (**c**). *p ≤ 0.05

Moreover hearts were isolated from adult PCSK9 knockout and control mice to asses left ventricular function. Mean LVDP (mmHg), dLVP/dt_max_ (mmHg/s) and dLVP/dt_min_ (mmHg/s) (Fig. 9) were significantly increased in hearts from PCSK9 knockout mice (*p ≤ 0.05).

**Fig. 10:**
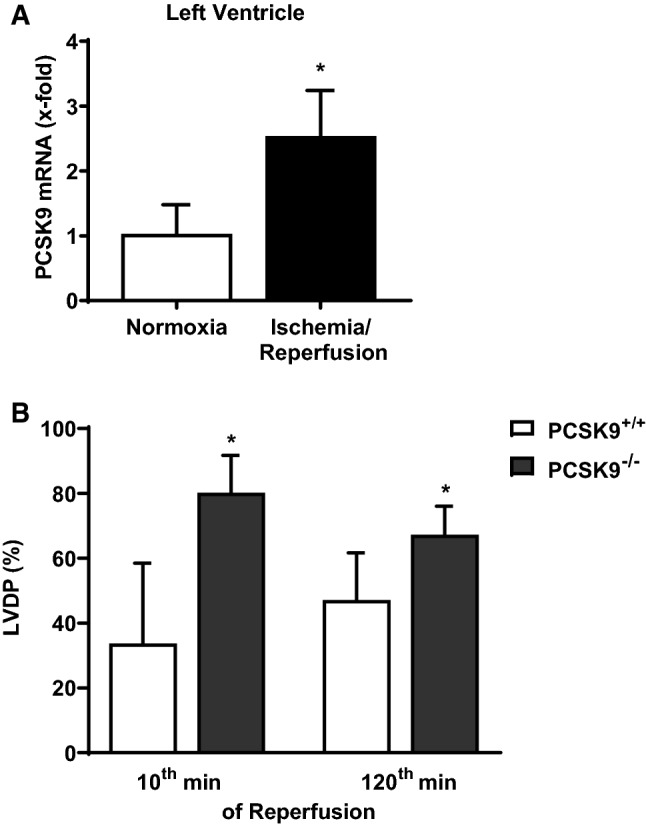
PCSK9 expression in ischemic mice hearts as well as left ventricular function of PCSK9 knockout hearts. **a** Hearts from C57BL6/JR mice were excised and exposed to 45 min of ischemia and 120 min reperfusion (I/R) or 165 min normoxia (Nx). PCSK9 mRNA expression of left ventricles was analyzed and normalized to the mean expression of B2M, HPRT and GAPDH. Nx: *n* = 10, I/R: *n* = 9. Statistical analysis was performed by unpaired *t*-test. *p ≤ 0.05. **b** Left ventricular developed pressure (LVDP) of the 10th minute as well as of the 120th minute of reperfusion from isolated hearts (PCSK9^−/−^- and PCSK9^+/+^-mice) was measured and normalized to the LVDP under basal conditions (given in %). *n* = 11–13. Statistical analysis was performed by unpaired *t*-test. *p ≤ 0.05. All data are mean ± SD

In addition PCSK9 mRNA was upregulated in left ventricles with ischemia/reperfusion (I/R) injury (ex vivo) (Fig. 10a). To evaluate the role of cardiac PCSK9 in myocardial I/R injury, we performed an I/R protocol with isolated hearts of PCSK9 knockout mice. LVDP (%) of PCSK9 knockout hearts was significantly increased during reperfusion (Fig. 10b) compared to the LVDP (%) of controls.

## Discussion

From our previous study it was already known that oxLDL induced the expression of PCSK9 in cardiomyocytes and reduced cardiomyocyte’ function in a PCSK9-dependent way [28]. To further study the mechanism behind this effect, the current study adds five new findings: (1) oxLDL reduces cell shortening in mice and rat cardiomyocytes. (2) OxLDL induces a paracrine effect of PCSK9 that than reduces cardiomyocyte cell shortening. (3) OxLDL leads to an increase in PCSK9 secretion that requires SURF-4 as in HEK293 cells [10]. (4) Antagonism of PCSK9 by monoclonal antibody (alirocumab) leads to an immediate increase in left ventricular function of isolated perfused hearts. (5) Hearts isolated from PCSK9 knockout mice show increased postischemic function. Therefore, cardiomyocyte-derived PCSK9 seems to directly reduce cardiomyocyte and cardiac function, respectively. The data suggest that the aforementioned superior effect of PCSK9 inhibition over cholesterol reduction performed by statins is supported by direct effects on cardiomyocyte-derived PCSK9.

Since its discovery in 2003 [2, 33] PCSK9 has emerged as an important target in the treatment of hyperlipidemia due to its role in hepatic LDL-clearance [6]. Apart from that distinct extrahepatic functions of PCSK9 have been described during the past years [12]. PCSK9 for example regulates the abundance of the epithelial sodium channel in the kidney [34] and is also expressed in pancreatic islets β cells where it contributes to glucose tolerance [8, 23]. Moreover studies report that patients with a loss of function mutation in PCSK9 show a cardiovascular protection that is higher than predicted by the associated reduction of LDL-C alone [4, 7, 19]. Therefore a possible direct negative effect of PCSK9 on the cardiovascular system, that is independent of its role in LDL-clearance, could be suggested.

Recently it was reported that PCSK9 plasma levels are associated with a reduced ejection fraction in patients suffering from ST-segment elevation myocardial infarction [24]. Furthermore a distinct function of cardiomyocyte-derived PCSK9 has only been reported for neonatal mouse cardiomyocytes, where under hypoxic conditions the release of PCSK9 determines autophagy in these cells [9]. Unfortunately this cell type is not directly comparable to terminally differentiated cardiomyocytes and autophagy not directly linked to mechanical function.

In our previous study we demonstrated that terminally differentiated cardiomyocytes isolated from rats constitutively express (mRNA, protein) and release PCSK9 [28]. Meanwhile the expression of PCSK9 in neonatal mouse cardiomyocytes as well as in ischemic mice hearts has been described [9]. In addition we reported that cardiomyocyte-derived PCSK9 is involved in the oxLDL-induced reduction of cardiomyocyte cell shortening [28]. In the current study we could reveal that the oxLDL-induced reduction of cardiomyocyte function requires PCSK9 because this effect was absent in PCSK9^−/−^- but present in PCSK9^+/+^-cardiomyocytes. With these experiments we could demonstrate the PCSK9 mediated oxLDL effect already in two different species (mouse, rat). In addition, we found no difference in basal function of cardiomyocytes from both strains. This supports the view that intracellular PCSK9 is not directly involved in cell function. In addition, only when PCSK9 expression is increased by oxLDL [28] and release is increased by oxLDL (present study) the threshold of extracellular PCSK9 is sufficient to reduce cardiomyocyte shortening. In addition the effect of oxidized LDL seems to depend on the oxidation-level of LDL. Furthermore, an oxidatively modified non-lipid plasma protein (MDA-HSA) had no effect on cardiomyocyte cell shortening.

The incubation of cardiomyocytes with supernatants collected from hPCSK9 overexpressing HepG2 cells, led to a significant decline of cardiac function that was absent under control conditions. Furthermore these findings were reproducible by incubation of cardiomyocytes with recombinant PCSK9. Therefore any unspecific effect of HepG2 supernatants on cell shortening can be excluded. The concentration of PCSK9 (200 ng/ml) used in this study to reduce cardiomyocyte function is comparable with the mean serum PCSK9 concentration found in rats [36] and humans [27].

Under oxLDL incubation PCSK9 was increased in a concentration-dependent manner. In addition we found that SURF-4, that has shown to be important for PCSK9 secretion [10, 32], is expressed in cardiomyocytes and that silencing of SURF-4 improves cardiomyocyte function when incubated with oxLDL. Furthermore we could block the oxLDL-induced reduction in cell shortening by alirocumab as well as Pep 2–8 while the inhibitors had no effect on basal cardiac function. This leads to the suggestion that oxLDL induces PCSK9 release and PCSK9 acts in an autocrine way to reduce cardiomyocyte function.

As exogenously added PCSK9 but also endogenous PCSK9 (rat PCSK9) has direct negative effects on cardiomyocyte function, the domain of PCSK9 that induces the reduction of cardiomyocyte cell shortening must lie in the conserved region between human and rat PCSK9. Overall, the total sequence identity of PCSK9 between these two species is about 77% [14]. Moreover the PCSK9 induced decline of cardiomyocyte function can be antagonized by Pep 2–8 which is known to inhibit the catalytic domain of PCSK9 that is responsible for LDL-R binding [16, 21, 38]. Therefore the catalytic domain might also be important for the abovementioned PCSK9 effect on cardiomyocyte function.

On the organ level we found that hearts isolated from PCSK9 knockout mice show increased basal cardiac function. In addition a cardiac release of PCSK9 was demonstrated under basal conditions in isolated rat hearts. Furthermore the antagonism of PCSK9 with alirocumab led to an immediate increase in left ventricular function. This is the first report about a direct effect of PCSK9 antagonism on basal cardiac function. Moreover, I/R of isolated mice hearts resulted in an increased expression of PCSK9 in left ventricles. In addition, hearts isolated from PCSK9 knockout mice showed increased postischemic left venctricular function and therefore an improved recovery after suffering from myocardial ischemia. So far an increased expression of PCSK9 in mice hearts, which have been subjected to acute ischemia/reperfusion (present study), myocardial ischemia for 1 week, as well as in human hearts with recent infarcts, was found [9]. Furthermore, it was shown that increased PCSK9 expression in hypoxic neonatal mouse cardiomyocytes is mediated by Hypoxia inducible factor-1 alpha (HIF-1α) [9]. Moreover a correlation between the amount of PCSK9 in the zone bordering the infarcted area and left ventricular function (1 week of left coronary artery occlusion) was observed [9]. In addition, PCSK9 inhibitors have been shown to improve cardiac function in rats with acute I/R injury when applied prior to ischemia [26]. Finally a clinical study reports a correlation between high PCSK9 plasma levels and a reduction of left ventricular ejection fraction in patients that suffered from ST-segment elevation myocardial infarction [24]. These studies demonstrate, similar to our findings, a positive effect of PCSK9 inhibition or genetic deletion on cardiac function after ischemia or I/R injury in vivo [9, 26]. Although in vivo models enable to study the effect of PCSK9 inhibition in a more complex way than ex vivo models they do not allow to distinguish between systemic and cardiac derived PCSK9.

Based on the experiments performed on isolated cardiomyocytes it could be hypothesized that also on the organ level, PCSK9 that is released from, e.g., cardiomyocytes acts in an autocrine way to reduce cardiac function. An increased PCSK9 secretion, that requires SURF-4, can be triggered by oxLDL. The exact mechanisms of PCSK9 mediated impaired cardiomyocyte/myocardial function and in this context binding/interaction partner of PCSK9 in cardiomyocytes have to be identified. Here a modulation of autophagy of cardiomyocytes mediated by PCSK9 could play a role [9, 37].

In conclusion, this study improves our current understanding about the relationship between high plasma levels of PCSK9 and cardiac function as well as our understanding about the mechanism by which drugs increasing cardiac expression of PCSK9 might affect cardiac function.

## Electronic supplementary material

Below is the link to the electronic supplementary material.

Supplementary file1 (DOCX 25 kb)
