# Supplementary material for: Autocrine effects of PCSK9 on cardiomyocytes
Source: Basic Res Cardiol. 2020 Nov 10;115(6):65. doi: 10.1007/s00395-020-00824-w (PMC7652747; doi:10.1007/s00395-020-00824-w)
Supplement: Supplementary file 1 — Supplementary file1 (DOCX 25 kb) [file 395_2020_824_MOESM1_ESM.docx]

**Supplement Figure 1:** Effect of malondialdehyde-modified human serum albumin (MDA-HSA) as well as LDL (with different levels of oxidation) on cell shortening of cardiomyocytes*.* Adult rat ventricular cardiomyocytes were cultured under serum free conditions and incubated with (A) serum albumin (10 µg/ml) and MDA-HSA (10 µg/ml)* as well as (B) LDL (20 µM), Low-oxLDL (20 µM) and High-oxLDL (20 µM) (in all other experiments we used “High-oxLDL”). After 24 h, load free cell shortening was determined (cells were paced at 2 Hz) and is expressed as ΔL/L (%). (A) Control=45, Albumin=17 and MDA-HSA=17cells (2-5 independent experiments with an intraassay variability of p > 0.05), (B) Control=81, LDL=90, Low-oxLDL=90 and High-oxLDL=90 cells (9-10 independent experiments with an intraassay variability of p > 0.05). Statistical analysis was performed by one-way ANOVA and Student-Newman-Keuls for post hoc analysis. * p ≤ 0.05. Data are mean ± SD

* Rahman, Mizanur; Steuer, Johnny; Gillgren, Peter; Végvári, Ákos; Liu, Anquan; Frostegård, Johan (2019): Malondialdehyde Conjugated With Albumin Induces Pro-Inflammatory Activation of T Cells Isolated From Human Atherosclerotic Plaques Both Directly and Via Dendritic Cell-Mediated Mechanism. In: *JACC. Basic to translational science* 4 (4), S. 480–494. DOI: 10.1016/j.jacbts.2019.03.009.
